# Crystal structure of 4-amino-1-(4-methyl­benz­yl)pyridinium bromide

**DOI:** 10.1107/S1600536814025343

**Published:** 2014-11-26

**Authors:** N. Sharmila, T. V. Sundar, A. Yasodha, A. Puratchikody, B. Sridhar

**Affiliations:** a Department of Physics, Shrimati Indira Gandhi College, Tiruchirappalli 620 002, Tamilnadu, India; b PG & Research Department of Physics, National College (Autonomous), Tiruchirappalli 620 001, Tamilnadu, India; c Department of Pharmaceutical Chemistry, PGP College of Pharmaceutical Science & Research Institute, Namakkal 637 207, India; d Drug Discovery and Developement Research Group, Department of Pharmaceutical Technology, Anna University Chennai, BIT Campus, Tiruchirappalli 620 024, Tamilnadu, India; e X-ray Crystallography Division, CSIR–Indian Institute of Chemical Technology, Uppal Road, Tarnaka, Hyderabad 500 607, Andhra Pradesh, India

**Keywords:** crystal structure, mol­ecular salt, pyridinium, bromide, hydrogen bonding

## Abstract

The title mol­ecular salt, C_13_H_15_N_2_^+^·Br^−^, crystallized with two independent ion pairs (*A* and *B*) in the asymmetric unit. In the cations, the planes of the pyridine and benzene rings are inclined to one another by 79.32 (8) and 82.30 (10)° in ion pairs *A* and *B*, respectively. In the crystal, the anions and cations are connected by N—H⋯Br hydrogen bonds, forming a centrosymmetric tetra­mer-like unit enclosing an *R*_8_^4^(16) ring motif. These units are linked *via* C—H⋯Br hydrogen bonds, forming a three-dimensional network.

## Related literature   

For the solid-phase synthesis of 1,3,5-tris­ubstituted pyridinium salts, see: Lago *et al.* (1998 ▶). For a review on quaternary pyridinium salts, see: Madaan & Tyagi (2008 ▶). For anti­microbial properties of quarternary amine derivatives, see: Thorsteinsson *et al.* (2003 ▶). For the pharmacological activity of 4-amino­pyridine compounds, see: Hansebout & Blight (1996 ▶); Kumar & Rao (2005 ▶). For the anti­microbial activity of 4-am­ino­pyridine compounds, see: Ilangovan *et al.* (2012 ▶); Sundararaman *et al.* (2013 ▶). For the crystal structures of related compounds, see: Seethalakshmi *et al.* (2006 ▶); Sundar *et al.* (2006*a* ▶,*b* ▶,*c* ▶).
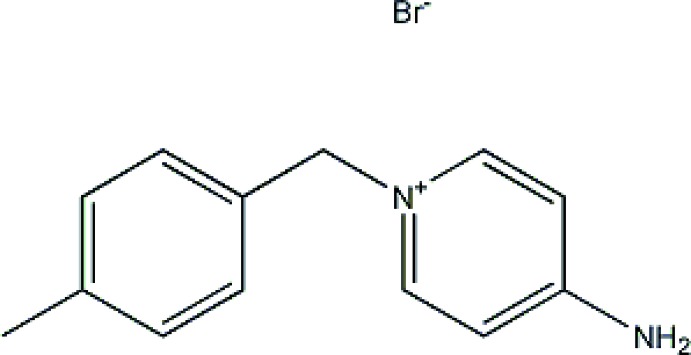


## Experimental   

### Crystal data  

C_13_H_15_N_2_^+^·Br^−^
*M* *_r_* = 279.18
Orthorhombic, 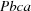

*a* = 10.5646 (6) Å
*b* = 18.7980 (11) Å
*c* = 26.9782 (16) Å
*V* = 5357.7 (5) Å^3^
*Z* = 16
Mo *K*α radiation
μ = 3.05 mm^−1^
*T* = 294 K
0.13 × 0.11 × 0.09 mm

### Data collection  

Bruker SMART CCD area-detector diffractometer
Absorption correction: multi-scan (*SADABS*; Bruker, 2001 ▶) *T*_min_ = 0.66, *T*_max_ = 0.79
59314 measured reflections
6400 independent reflections
4055 reflections with *I* > 2σ(*I*)
*R*_int_ = 0.057

### Refinement  

*R*[*F* ^2^ > 2σ(*F* ^2^)] = 0.039
*wR*(*F* ^2^) = 0.110
*S* = 1.01
6400 reflections
307 parameters
H atoms treated by a mixture of independent and constrained refinement
Δρ_max_ = 0.66 e Å^−3^
Δρ_min_ = −0.36 e Å^−3^

### 

Data collection: *SMART* (Bruker, 2001 ▶); cell refinement: *SAINT* (Bruker, 2001 ▶); data reduction: *SAINT*; program(s) used to solve structure: *SHELXS97* (Sheldrick, 2008 ▶); program(s) used to refine structure: *SHELXL2013* (Sheldrick, 2008 ▶); molecular graphics: *ORTEP-3 for Windows* (Farrugia, 2012 ▶) and *Mercury* (Macrae *et al.*, 2008 ▶); software used to prepare material for publication: *WinGX* (Farrugia, 2012 ▶) and *PLATON* (Spek, 2009 ▶).

## Supplementary Material

Crystal structure: contains datablock(s) I, New_Global_Publ_Block. DOI: 10.1107/S1600536814025343/su5023sup1.cif

Structure factors: contains datablock(s) I. DOI: 10.1107/S1600536814025343/su5023Isup2.hkl

Supporting information file. DOI: 10.1107/S1600536814025343/su5023Isup3.cml

**Fig. 1.**
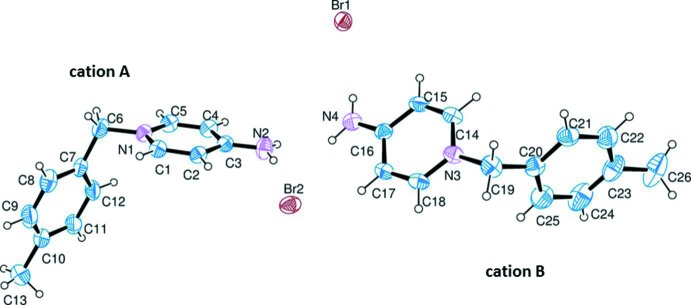
The molecular structure of the two independent cations and anions of the title molecular salt, with atom labelling. Displacement ellipsoids are drawn at the 30% probability level.

. DOI: 10.1107/S1600536814025343/su5023fig1.tif
The mol­ecular structure of the two independent cations and anions of the title mol­ecular salt, with atom labelling. Displacement ellipsoids are drawn at the 30% probability level.

**Fig. 2.**
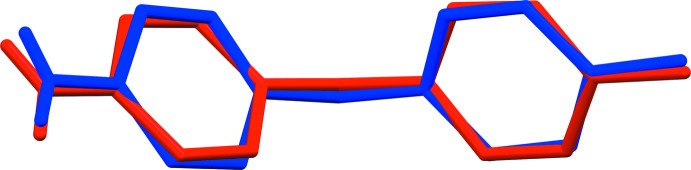
A view of the molecular overlay of the two independent cations (cation A blue, cation B red).

. DOI: 10.1107/S1600536814025343/su5023fig2.tif
A view of the mol­ecular overlay of the two independent cations (cation A blue, cation B red).

**Fig. 3.**
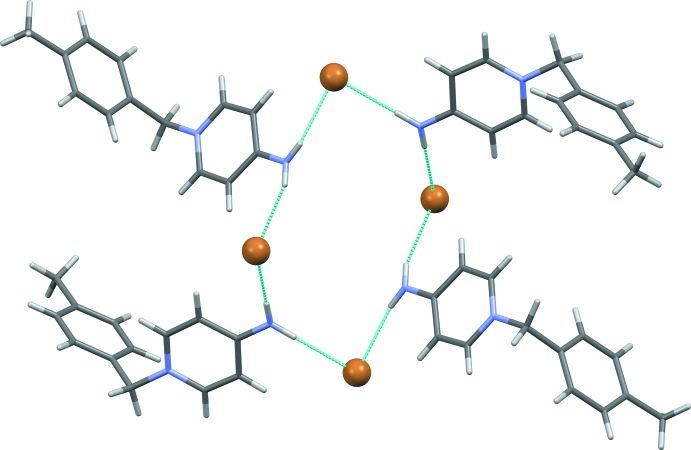
A view of the tetramer-like unit enclosing an R84(16) ring motif in the crystal structure of the title salt. The N–H···Br hydrogen bonds are shown as dashed lines (see Table 1 for details).

**Fig. 4.**
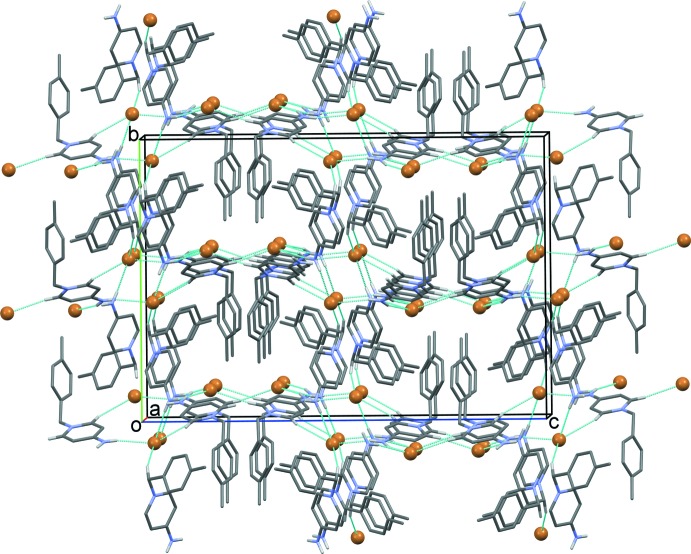
Crystal packing of the title compound, viewed along the a axis, showing the N–H···Br and C-H···Br hydrogen bonds as dashed lines (see Table 1 for details; H atoms not involved in these interactions have been omitted for clarity).

. DOI: 10.1107/S1600536814025343/su5023fig3.tif
A view of the tetra­mer-like unit enclosing an 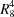
(16) ring motif in the crystal structure of the title salt. The N–H⋯Br hydrogen bonds are shown as dashed lines (see Table 1 for details).

a . DOI: 10.1107/S1600536814025343/su5023fig4.tif
Crystal packing of the title compound, viewed along the *a* axis, showing the N–H⋯Br and C-H⋯Br hydrogen bonds as dashed lines (see Table 1 for details; H atoms not involved in these inter­actions have been omitted for clarity).

CCDC reference: 1034948

Additional supporting information:  crystallographic information; 3D view; checkCIF report

## Figures and Tables

**Table 1:** Hydrogen-bond geometry (, )

*D*H*A*	*D*H	H*A*	*D* *A*	*D*H*A*
N2H1*N*2Br2	0.80(3)	2.58(3)	3.367(4)	169(3)
N4H1*N*4Br2	0.87(3)	2.52(4)	3.375(3)	166(3)
N4H2*N*4Br1	0.80(3)	2.57(3)	3.363(3)	169(3)
N2H2*N*2Br1^i^	0.79(3)	2.65(3)	3.410(4)	161(3)
C6H6*B*Br1^ii^	0.97	2.87	3.745(3)	151
C14H14Br1^iii^	0.93	2.86	3.602(3)	138
C18H18Br2^iv^	0.93	2.74	3.656(3)	169

Symmetry codes: (i) 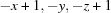; (ii) 
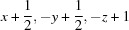
; (iii) 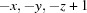; (iv) 
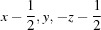
.

## supplementary crystallographic information

### S1. Comment

Quarternary amine derivatives have been reported to possess antimicrobial properties (Thorsteinsson *et al.*, 2003). The use of 4-aminopyridine derivatives are reported to be useful for the treatment of a peripheral nervous system demyelinating disease selected from Guillain-Barre syndrome diabetes mellitus and hereditary sensory-motor neuropathy. Literature information revealed that the use of 4-aminopyridine had reduced chronic pain and spasticity in spinal cord injured patients (Hansebout *et al.*, 1996). The crystal structures of a large number of pyridinium derivatives have been reported (Seethalakshmi *et al.*, 2006; Sundar *et al.*, 2006*a*,b,c) and their antimicrobial activities have also been reported (Ilangovan *et al.*, 2012; Sundararaman *et al.*, 2013).

The molecular structure of the two independent cations and anions of the title molecular salt is illustrated in Fig. 1. In the cations, the pyridine and benzene rings are inclined to one another by 79.32 (8)° in molecule A and by 82.30 (10)° in molecule B (Fig. 2). These values are similar to those observed in the crystal structures of some related compounds (Seethalakshmi *et al.*, 2006; Sundar *et al.*, 2006*a*,b,c). The overlap of the two cations in the title compound is illustrated in Fig. 2.

In the crystal, the cations and anions are linked *via* N-H···Br hydrogen bonds forming a tetramer-like unit enclosing an *R*_8_^4^(16) ring motif (Table 1 and Fig. 3). These units are linked *via* C-H···Br hydrogen bonds forming a three-dimensional framework (Table 1 and Fig. 4).

### S2. Experimental

A mixture of 4-methylbenzylbromide (0.1 mol) and 4-aminopyridine (0.1 mol) in dry acetone (50 ml) was stirred at room temperature for 2–12 h. The pale yellow solid that separated was filtered off, washed with toluene, dried under vacuum to give the stable title molecular salt. It was recrystallized from chloroform-acetone (1:1, *v*/*v*), giving thin yellow plate-like crystals.

### S3. Refinement

The N-bound H atoms were located in a difference Fourier map and freely refined. The C-bound H atoms were included in calculated positions and treated as riding atoms: C-H = 0.93 - 0.97 Å with U_iso_(H) = 1.5U_eq_(C) for methyl H atoms and = 1.2U_eq_(C) for other H atoms.

### Crystal data

**Table:**

C_13_H_15_N_2_^+^·Br^−^	*D*_x_ = 1.384 Mg m^−^^3^
*M**_r_* = 279.18	Melting point: 495 K
Orthorhombic, *P**b**c**a*	Mo *K*α radiation, λ = 0.71073 Å
*a* = 10.5646 (6) Å	Cell parameters from 5959 reflections
*b* = 18.7980 (11) Å	θ = 2.3–24.2°
*c* = 26.9782 (16) Å	µ = 3.05 mm^−^^1^
*V* = 5357.7 (5) Å^3^	*T* = 294 K
*Z* = 16	Block, yellow
*F*(000) = 2272	0.13 × 0.11 × 0.09 mm

### Data collection

**Table:**

Bruker SMART CCD area-detector diffractometer	6400 independent reflections
Radiation source: fine focus sealed tube	4055 reflections with *I* > 2σ(*I*)
Graphite monochromator	*R*_int_ = 0.057
ω scans	θ_max_ = 28.0°, θ_min_ = 2.2°
Absorption correction: multi-scan (*SADABS*; Bruker, 2001)	*h* = −13→13
*T*_min_ = 0.66, *T*_max_ = 0.79	*k* = −24→24
59314 measured reflections	*l* = −35→35

### Refinement

**Table:**

Refinement on *F*^2^	Primary atom site location: structure-invariant direct methods
Least-squares matrix: full	Secondary atom site location: difference Fourier map
*R*[*F*^2^ > 2σ(*F*^2^)] = 0.039	Hydrogen site location: mixed
*wR*(*F*^2^) = 0.110	H atoms treated by a mixture of independent and constrained refinement
*S* = 1.01	*w* = 1/[σ^2^(*F*_o_^2^) + (0.0541*P*)^2^ + 1.4559*P*] where *P* = (*F*_o_^2^ + 2*F*_c_^2^)/3
6400 reflections	(Δ/σ)_max_ = 0.003
307 parameters	Δρ_max_ = 0.66 e Å^−^^3^
0 restraints	Δρ_min_ = −0.36 e Å^−^^3^

### Special details

**Table:**

Geometry. All e.s.d.'s (except the e.s.d. in the dihedral angle between two l.s. planes) are estimated using the full covariance matrix. The cell e.s.d.'s are taken into account individually in the estimation of e.s.d.'s in distances, angles and torsion angles; correlations between e.s.d.'s in cell parameters are only used when they are defined by crystal symmetry. An approximate (isotropic) treatment of cell e.s.d.'s is used for estimating e.s.d.'s involving l.s. planes.

### Fractional atomic coordinates and isotropic or equivalent isotropic displacement parameters (Å^2^)

**Table:**

	*x*	*y*	*z*	*U*_iso_*/*U*_eq_	
Br1	0.20297 (3)	0.08005 (2)	0.52655 (2)	0.06129 (12)	
Br2	0.49073 (3)	0.11126 (2)	0.33059 (2)	0.07094 (13)	
N1	0.8934 (2)	0.23701 (13)	0.47182 (8)	0.0526 (6)	
N2	0.6590 (3)	0.07346 (18)	0.43327 (13)	0.0690 (8)	
N3	−0.0725 (2)	0.02494 (12)	0.31892 (8)	0.0527 (6)	
N4	0.2390 (3)	0.07293 (16)	0.40281 (12)	0.0643 (7)	
C1	0.8056 (3)	0.24614 (16)	0.43621 (11)	0.0545 (7)	
H1	0.7986	0.2902	0.4208	0.065*	
C2	0.7276 (3)	0.19293 (15)	0.42224 (10)	0.0512 (7)	
H2	0.6685	0.2005	0.3972	0.061*	
C3	0.7354 (3)	0.12630 (15)	0.44526 (11)	0.0512 (7)	
C4	0.8294 (3)	0.11806 (17)	0.48171 (11)	0.0579 (7)	
H4	0.8399	0.0744	0.4974	0.069*	
C5	0.9042 (3)	0.17331 (16)	0.49393 (11)	0.0571 (7)	
H5	0.9652	0.1672	0.5184	0.069*	
C6	0.9791 (3)	0.29613 (17)	0.48495 (13)	0.0663 (9)	
H6A	1.0192	0.2859	0.5165	0.080*	
H6B	0.9301	0.3394	0.4887	0.080*	
C7	1.0797 (3)	0.30778 (15)	0.44639 (11)	0.0564 (7)	
C8	1.0930 (3)	0.37270 (18)	0.42366 (14)	0.0748 (10)	
H8	1.0377	0.4095	0.4314	0.090*	
C9	1.1883 (4)	0.3837 (2)	0.38927 (15)	0.0849 (11)	
H9	1.1959	0.4282	0.3744	0.102*	
C10	1.2714 (3)	0.3313 (2)	0.37666 (13)	0.0753 (10)	
C11	1.2567 (3)	0.26595 (19)	0.39924 (14)	0.0767 (10)	
H11	1.3122	0.2292	0.3914	0.092*	
C12	1.1617 (3)	0.25412 (17)	0.43303 (13)	0.0684 (9)	
H12	1.1527	0.2092	0.4471	0.082*	
C13	1.3778 (5)	0.3440 (3)	0.34078 (15)	0.1151 (16)	
H13A	1.4552	0.3511	0.3588	0.173*	
H13B	1.3598	0.3855	0.3213	0.173*	
H13C	1.3866	0.3035	0.3194	0.173*	
C14	−0.0710 (3)	0.01177 (16)	0.36826 (10)	0.0579 (7)	
H14	−0.1418	−0.0089	0.3828	0.069*	
C15	0.0289 (3)	0.02757 (16)	0.39685 (10)	0.0580 (7)	
H15	0.0259	0.0183	0.4307	0.070*	
C16	0.1381 (3)	0.05795 (14)	0.37614 (10)	0.0503 (7)	
C17	0.1351 (3)	0.07113 (16)	0.32494 (10)	0.0579 (7)	
H17	0.2052	0.0911	0.3093	0.070*	
C18	0.0305 (3)	0.05486 (16)	0.29817 (11)	0.0576 (7)	
H18	0.0298	0.0647	0.2644	0.069*	
C19	−0.1864 (3)	0.00951 (18)	0.28897 (12)	0.0677 (9)	
H19A	−0.1702	0.0227	0.2548	0.081*	
H19B	−0.2560	0.0385	0.3009	0.081*	
C20	−0.2245 (3)	−0.06752 (17)	0.29092 (11)	0.0585 (8)	
C21	−0.3357 (3)	−0.08812 (19)	0.31320 (13)	0.0685 (9)	
H21	−0.3878	−0.0542	0.3279	0.082*	
C22	−0.3705 (4)	−0.1588 (2)	0.31388 (13)	0.0787 (10)	
H22	−0.4461	−0.1716	0.3291	0.094*	
C23	−0.2974 (4)	−0.2105 (2)	0.29295 (15)	0.0845 (11)	
C24	−0.1864 (4)	−0.1896 (2)	0.27187 (18)	0.1021 (14)	
H24	−0.1337	−0.2239	0.2580	0.122*	
C25	−0.1498 (4)	−0.1197 (2)	0.27045 (16)	0.0883 (12)	
H25	−0.0736	−0.1074	0.2555	0.106*	
C26	−0.3374 (5)	−0.2880 (2)	0.29280 (19)	0.1277 (19)	
H26A	−0.4078	−0.2944	0.3149	0.192*	
H26B	−0.2680	−0.3171	0.3036	0.192*	
H26C	−0.3618	−0.3017	0.2599	0.192*	
H1N2	0.613 (3)	0.0776 (16)	0.4100 (12)	0.057 (10)*	
H1N4	0.305 (3)	0.0899 (17)	0.3877 (13)	0.074 (11)*	
H2N4	0.230 (3)	0.0685 (15)	0.4322 (13)	0.055 (9)*	
H2N2	0.674 (3)	0.0357 (18)	0.4451 (12)	0.059 (10)*	

### Atomic displacement parameters (Å^2^)

**Table:**

	*U*^11^	*U*^22^	*U*^33^	*U*^12^	*U*^13^	*U*^23^
Br1	0.0765 (2)	0.05288 (19)	0.05445 (19)	−0.00899 (15)	−0.00301 (14)	0.00835 (13)
Br2	0.0549 (2)	0.1048 (3)	0.05311 (19)	0.00089 (17)	−0.00372 (14)	−0.01363 (17)
N1	0.0399 (12)	0.0587 (15)	0.0591 (14)	0.0007 (11)	−0.0039 (10)	−0.0095 (11)
N2	0.0686 (19)	0.0570 (19)	0.081 (2)	−0.0046 (15)	−0.0201 (17)	0.0050 (16)
N3	0.0518 (13)	0.0577 (14)	0.0486 (13)	0.0059 (11)	0.0006 (11)	0.0076 (11)
N4	0.073 (2)	0.0718 (19)	0.0485 (16)	−0.0098 (15)	0.0005 (15)	−0.0026 (14)
C1	0.0522 (16)	0.0535 (17)	0.0578 (17)	0.0041 (14)	−0.0007 (13)	−0.0003 (13)
C2	0.0463 (15)	0.0568 (17)	0.0506 (15)	0.0048 (13)	−0.0067 (12)	−0.0039 (13)
C3	0.0441 (15)	0.0553 (18)	0.0542 (16)	0.0020 (13)	−0.0001 (12)	−0.0051 (13)
C4	0.0562 (17)	0.0577 (19)	0.0596 (18)	0.0088 (14)	−0.0068 (14)	0.0011 (14)
C5	0.0481 (16)	0.066 (2)	0.0575 (17)	0.0103 (15)	−0.0097 (13)	−0.0048 (15)
C6	0.0542 (18)	0.066 (2)	0.078 (2)	−0.0051 (15)	−0.0061 (15)	−0.0222 (17)
C7	0.0517 (17)	0.0467 (17)	0.0709 (19)	−0.0067 (13)	−0.0070 (14)	−0.0114 (14)
C8	0.072 (2)	0.0532 (19)	0.099 (3)	0.0015 (17)	−0.014 (2)	−0.0066 (18)
C9	0.102 (3)	0.062 (2)	0.090 (3)	−0.025 (2)	−0.012 (2)	0.011 (2)
C10	0.083 (2)	0.072 (2)	0.071 (2)	−0.026 (2)	0.0015 (18)	−0.0113 (18)
C11	0.071 (2)	0.063 (2)	0.096 (3)	−0.0114 (18)	0.0198 (19)	−0.0146 (19)
C12	0.070 (2)	0.0451 (17)	0.090 (2)	−0.0081 (15)	0.0087 (18)	−0.0014 (16)
C13	0.126 (4)	0.122 (4)	0.097 (3)	−0.054 (3)	0.029 (3)	−0.012 (3)
C14	0.0540 (17)	0.0681 (19)	0.0515 (16)	0.0025 (15)	0.0119 (14)	0.0086 (14)
C15	0.070 (2)	0.0645 (19)	0.0397 (14)	0.0002 (15)	0.0102 (14)	0.0036 (13)
C16	0.0550 (17)	0.0477 (16)	0.0481 (16)	0.0051 (13)	0.0061 (13)	−0.0061 (12)
C17	0.0582 (18)	0.067 (2)	0.0490 (16)	−0.0052 (15)	0.0103 (14)	0.0033 (14)
C18	0.0652 (19)	0.0655 (19)	0.0422 (15)	0.0035 (15)	0.0077 (13)	0.0094 (13)
C19	0.0534 (17)	0.083 (2)	0.0666 (19)	0.0043 (16)	−0.0105 (15)	0.0140 (17)
C20	0.0488 (17)	0.072 (2)	0.0544 (17)	0.0084 (14)	−0.0113 (13)	−0.0025 (14)
C21	0.067 (2)	0.074 (2)	0.065 (2)	0.0093 (17)	0.0011 (16)	−0.0046 (17)
C22	0.085 (2)	0.080 (3)	0.071 (2)	−0.011 (2)	−0.0082 (19)	0.0051 (19)
C23	0.098 (3)	0.072 (2)	0.084 (3)	0.006 (2)	−0.045 (2)	−0.005 (2)
C24	0.087 (3)	0.085 (3)	0.134 (4)	0.029 (2)	−0.019 (3)	−0.043 (3)
C25	0.059 (2)	0.103 (3)	0.102 (3)	0.010 (2)	0.001 (2)	−0.028 (2)
C26	0.166 (5)	0.074 (3)	0.143 (4)	−0.007 (3)	−0.074 (4)	−0.017 (3)

### Geometric parameters (Å, º)

**Table:**

N1—C5	1.343 (4)	C11—C12	1.374 (4)
N1—C1	1.346 (3)	C11—H11	0.9300
N1—C6	1.477 (4)	C12—H12	0.9300
N2—C3	1.320 (4)	C13—H13A	0.9600
N2—H1N2	0.80 (3)	C13—H13B	0.9600
N2—H2N2	0.79 (3)	C13—H13C	0.9600
N3—C18	1.347 (4)	C14—C15	1.341 (4)
N3—C14	1.354 (3)	C14—H14	0.9300
N3—C19	1.478 (4)	C15—C16	1.403 (4)
N4—C16	1.316 (4)	C15—H15	0.9300
N4—H1N4	0.87 (3)	C16—C17	1.404 (4)
N4—H2N4	0.80 (3)	C17—C18	1.355 (4)
C1—C2	1.350 (4)	C17—H17	0.9300
C1—H1	0.9300	C18—H18	0.9300
C2—C3	1.400 (4)	C19—C20	1.504 (4)
C2—H2	0.9300	C19—H19A	0.9700
C3—C4	1.406 (4)	C19—H19B	0.9700
C4—C5	1.346 (4)	C20—C21	1.375 (5)
C4—H4	0.9300	C20—C25	1.375 (5)
C5—H5	0.9300	C21—C22	1.378 (5)
C6—C7	1.503 (4)	C21—H21	0.9300
C6—H6A	0.9700	C22—C23	1.363 (5)
C6—H6B	0.9700	C22—H22	0.9300
C7—C8	1.373 (4)	C23—C24	1.361 (6)
C7—C12	1.378 (4)	C23—C26	1.519 (6)
C8—C9	1.385 (5)	C24—C25	1.370 (6)
C8—H8	0.9300	C24—H24	0.9300
C9—C10	1.363 (5)	C25—H25	0.9300
C9—H9	0.9300	C26—H26A	0.9600
C10—C11	1.380 (5)	C26—H26B	0.9600
C10—C13	1.503 (5)	C26—H26C	0.9600
			
C5—N1—C1	119.3 (2)	C10—C13—H13A	109.5
C5—N1—C6	120.8 (2)	C10—C13—H13B	109.5
C1—N1—C6	119.8 (3)	H13A—C13—H13B	109.5
C3—N2—H1N2	119 (2)	C10—C13—H13C	109.5
C3—N2—H2N2	117 (2)	H13A—C13—H13C	109.5
H1N2—N2—H2N2	122 (3)	H13B—C13—H13C	109.5
C18—N3—C14	118.4 (2)	C15—C14—N3	122.3 (3)
C18—N3—C19	120.8 (2)	C15—C14—H14	118.9
C14—N3—C19	120.7 (2)	N3—C14—H14	118.9
C16—N4—H1N4	118 (2)	C14—C15—C16	120.6 (3)
C16—N4—H2N4	115 (2)	C14—C15—H15	119.7
H1N4—N4—H2N4	127 (3)	C16—C15—H15	119.7
N1—C1—C2	121.7 (3)	N4—C16—C15	122.4 (3)
N1—C1—H1	119.2	N4—C16—C17	121.2 (3)
C2—C1—H1	119.2	C15—C16—C17	116.4 (3)
C1—C2—C3	120.2 (3)	C18—C17—C16	120.2 (3)
C1—C2—H2	119.9	C18—C17—H17	119.9
C3—C2—H2	119.9	C16—C17—H17	119.9
N2—C3—C2	121.9 (3)	N3—C18—C17	122.1 (3)
N2—C3—C4	121.4 (3)	N3—C18—H18	118.9
C2—C3—C4	116.8 (3)	C17—C18—H18	118.9
C5—C4—C3	120.1 (3)	N3—C19—C20	112.8 (2)
C5—C4—H4	120.0	N3—C19—H19A	109.0
C3—C4—H4	120.0	C20—C19—H19A	109.0
N1—C5—C4	122.0 (3)	N3—C19—H19B	109.0
N1—C5—H5	119.0	C20—C19—H19B	109.0
C4—C5—H5	119.0	H19A—C19—H19B	107.8
N1—C6—C7	112.2 (2)	C21—C20—C25	117.7 (3)
N1—C6—H6A	109.2	C21—C20—C19	121.0 (3)
C7—C6—H6A	109.2	C25—C20—C19	121.3 (3)
N1—C6—H6B	109.2	C20—C21—C22	120.3 (3)
C7—C6—H6B	109.2	C20—C21—H21	119.8
H6A—C6—H6B	107.9	C22—C21—H21	119.8
C8—C7—C12	118.0 (3)	C23—C22—C21	122.0 (4)
C8—C7—C6	120.7 (3)	C23—C22—H22	119.0
C12—C7—C6	121.3 (3)	C21—C22—H22	119.0
C7—C8—C9	120.5 (3)	C24—C23—C22	117.1 (4)
C7—C8—H8	119.8	C24—C23—C26	121.1 (4)
C9—C8—H8	119.8	C22—C23—C26	121.8 (4)
C10—C9—C8	121.8 (3)	C23—C24—C25	122.1 (4)
C10—C9—H9	119.1	C23—C24—H24	119.0
C8—C9—H9	119.1	C25—C24—H24	119.0
C9—C10—C11	117.5 (3)	C24—C25—C20	120.7 (4)
C9—C10—C13	121.8 (4)	C24—C25—H25	119.6
C11—C10—C13	120.7 (4)	C20—C25—H25	119.6
C12—C11—C10	121.3 (3)	C23—C26—H26A	109.5
C12—C11—H11	119.4	C23—C26—H26B	109.5
C10—C11—H11	119.4	H26A—C26—H26B	109.5
C11—C12—C7	121.0 (3)	C23—C26—H26C	109.5
C11—C12—H12	119.5	H26A—C26—H26C	109.5
C7—C12—H12	119.5	H26B—C26—H26C	109.5
			
C5—N1—C1—C2	0.3 (4)	C18—N3—C14—C15	0.1 (4)
C6—N1—C1—C2	178.3 (3)	C19—N3—C14—C15	−177.5 (3)
N1—C1—C2—C3	0.8 (4)	N3—C14—C15—C16	−0.9 (5)
C1—C2—C3—N2	178.6 (3)	C14—C15—C16—N4	−178.7 (3)
C1—C2—C3—C4	−1.8 (4)	C14—C15—C16—C17	0.7 (4)
N2—C3—C4—C5	−178.6 (3)	N4—C16—C17—C18	179.6 (3)
C2—C3—C4—C5	1.8 (4)	C15—C16—C17—C18	0.2 (4)
C1—N1—C5—C4	−0.3 (4)	C14—N3—C18—C17	0.9 (4)
C6—N1—C5—C4	−178.2 (3)	C19—N3—C18—C17	178.4 (3)
C3—C4—C5—N1	−0.8 (5)	C16—C17—C18—N3	−1.0 (5)
C5—N1—C6—C7	103.4 (3)	C18—N3—C19—C20	123.3 (3)
C1—N1—C6—C7	−74.6 (3)	C14—N3—C19—C20	−59.2 (4)
N1—C6—C7—C8	122.9 (3)	N3—C19—C20—C21	113.3 (3)
N1—C6—C7—C12	−57.6 (4)	N3—C19—C20—C25	−66.9 (4)
C12—C7—C8—C9	−1.5 (5)	C25—C20—C21—C22	−1.1 (5)
C6—C7—C8—C9	178.0 (3)	C19—C20—C21—C22	178.8 (3)
C7—C8—C9—C10	0.2 (6)	C20—C21—C22—C23	0.1 (5)
C8—C9—C10—C11	0.4 (6)	C21—C22—C23—C24	1.2 (5)
C8—C9—C10—C13	−177.7 (4)	C21—C22—C23—C26	−178.6 (3)
C9—C10—C11—C12	0.2 (5)	C22—C23—C24—C25	−1.5 (6)
C13—C10—C11—C12	178.4 (4)	C26—C23—C24—C25	178.3 (4)
C10—C11—C12—C7	−1.6 (5)	C23—C24—C25—C20	0.5 (7)
C8—C7—C12—C11	2.2 (5)	C21—C20—C25—C24	0.8 (6)
C6—C7—C12—C11	−177.3 (3)	C19—C20—C25—C24	−179.1 (4)

### Hydrogen-bond geometry (Å, º)

**Table:**

*D*—H···*A*	*D*—H	H···*A*	*D*···*A*	*D*—H···*A*
N2—H1*N*2···Br2	0.80 (3)	2.58 (3)	3.367 (4)	169 (3)
N4—H1*N*4···Br2	0.87 (3)	2.52 (4)	3.375 (3)	166 (3)
N4—H2*N*4···Br1	0.80 (3)	2.57 (3)	3.363 (3)	169 (3)
N2—H2*N*2···Br1^i^	0.79 (3)	2.65 (3)	3.410 (4)	161 (3)
C6—H6*B*···Br1^ii^	0.97	2.87	3.745 (3)	151
C14—H14···Br1^iii^	0.93	2.86	3.602 (3)	138
C18—H18···Br2^iv^	0.93	2.74	3.656 (3)	169

Symmetry codes: (i) −*x*+1, −*y*, −*z*+1; (ii) *x*+1/2, −*y*+1/2, −*z*+1; (iii) −*x*, −*y*, −*z*+1; (iv) *x*−1/2, *y*, −*z*−1/2.
