# Supplementary figures and images for: Crystal structure of 4-amino-1-(4-methyl­benz­yl)pyridinium bromide
Source: Acta Crystallogr Sect E Struct Rep Online. 2014 Nov 26;70(Pt 12):o1293–4. doi: 10.1107/S1600536814025343 (PMC4257457; doi:10.1107/S1600536814025343)

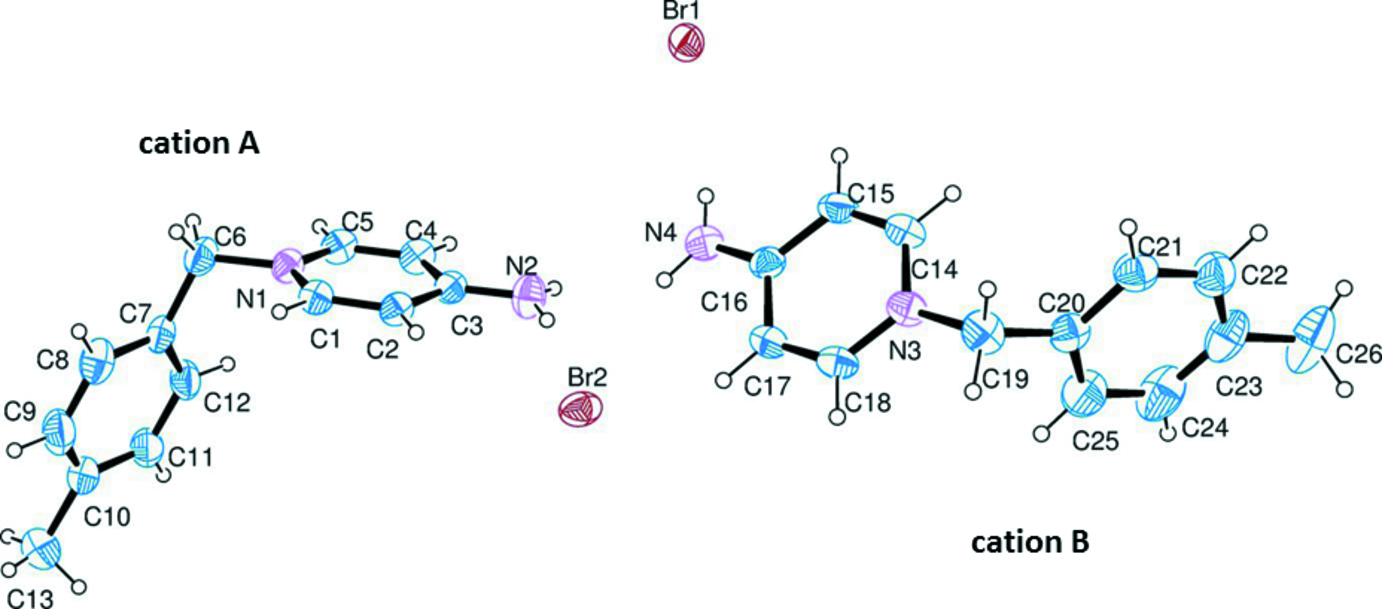

Supplement: Supplementary file 4 [file e-70-o1293-fig1.tif]

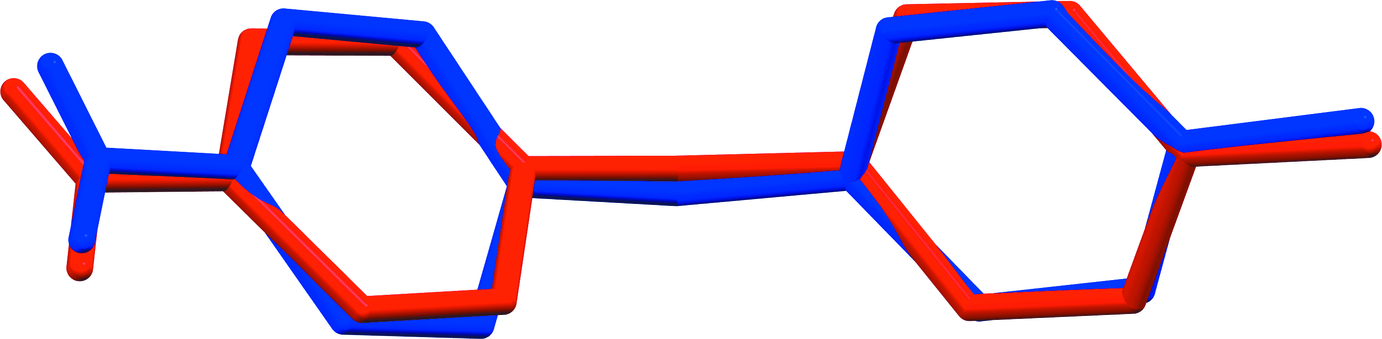

Supplement: Supplementary file 5 [file e-70-o1293-fig2.tif]

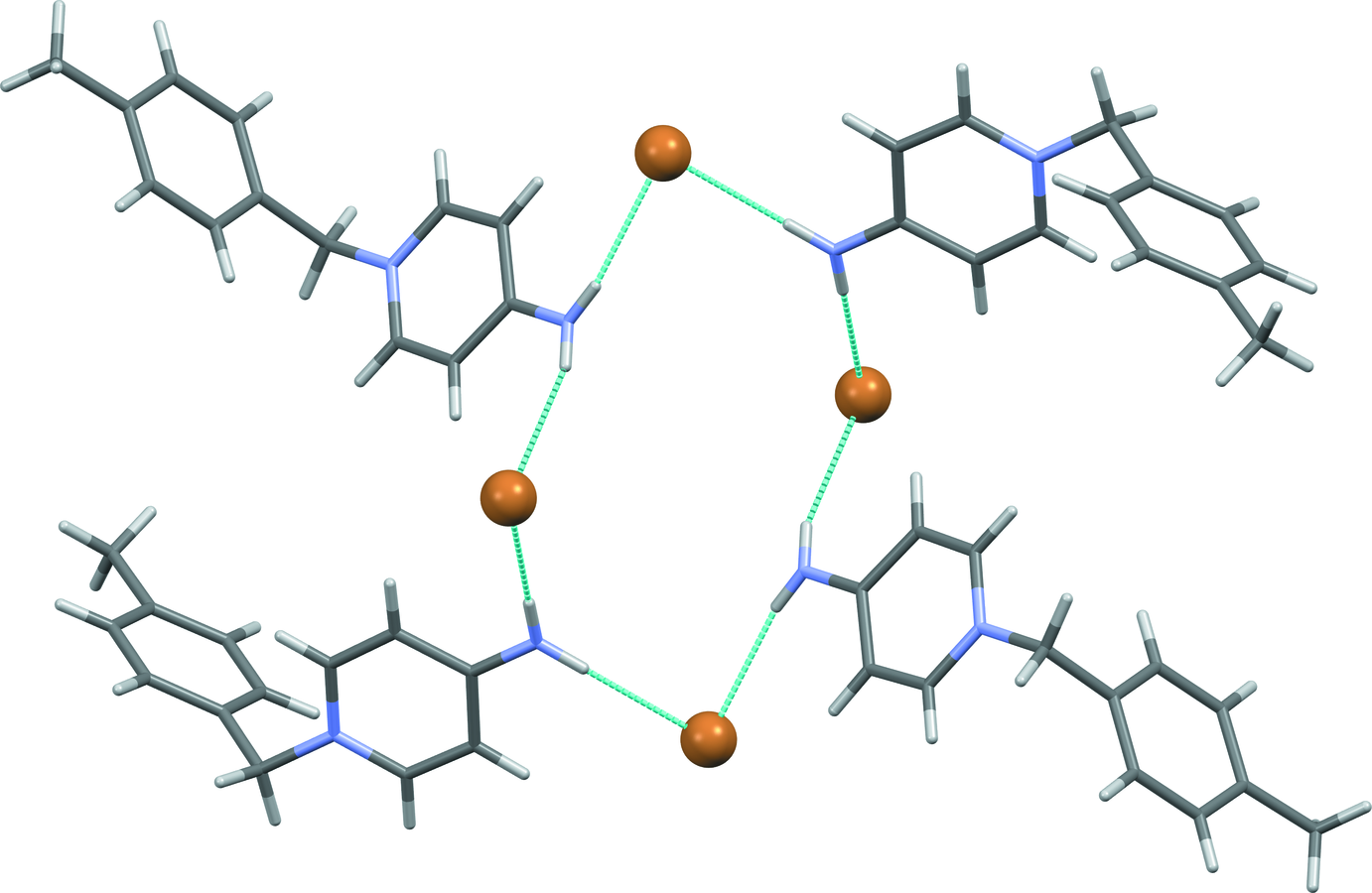

Supplement: Supplementary file 6 [file e-70-o1293-fig3.tif]

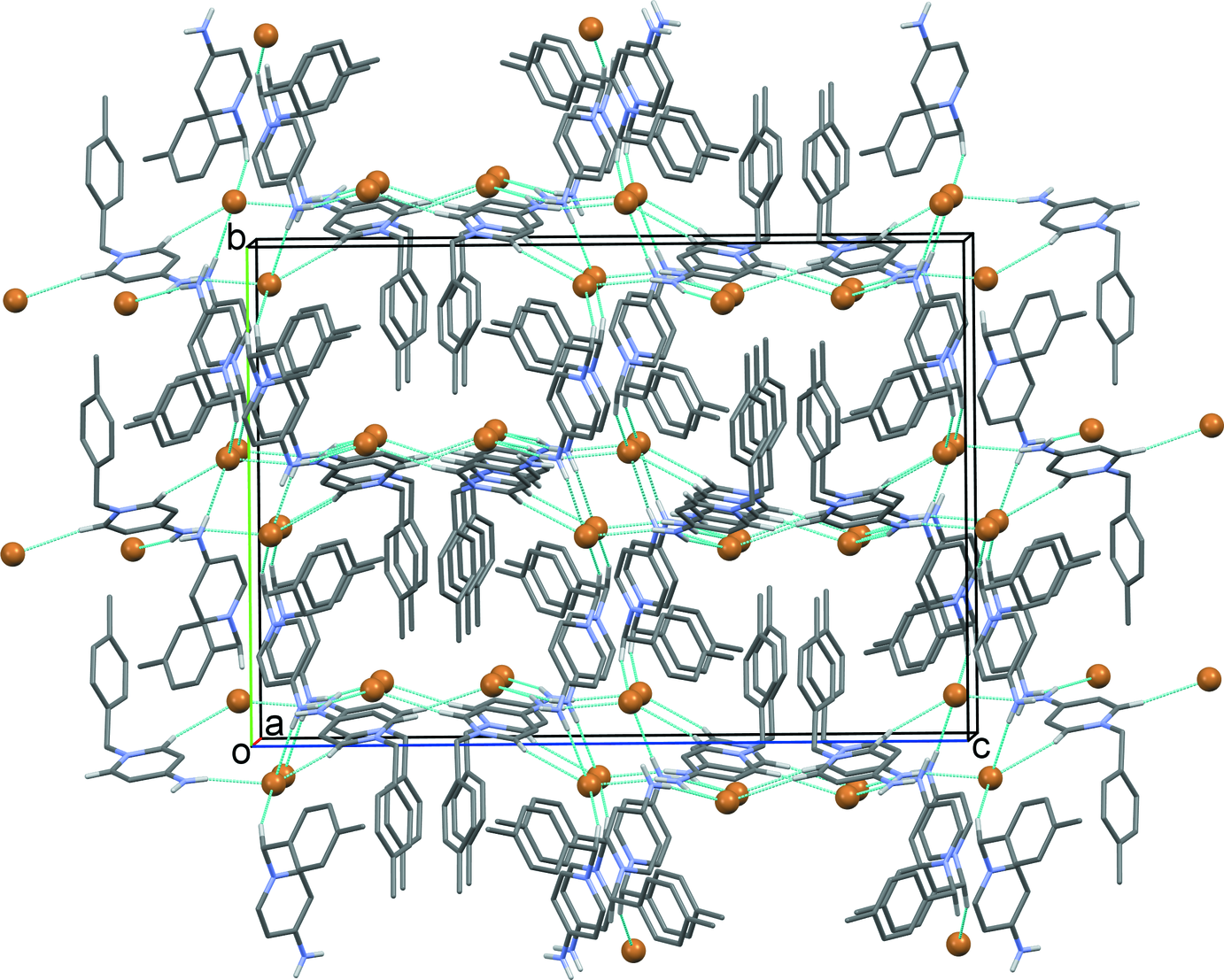

Supplement: Supplementary file 7 [file e-70-o1293-fig4.tif]
